# Ivabradine Attenuates Experimental Hepatic Fibrosis by Modulating Inflammatory and Apoptotic Signaling Pathways

**DOI:** 10.3390/ph19030504

**Published:** 2026-03-19

**Authors:** Salman H. Alotaibi, Mahmoud M. Samaha, Manar G. Helal, Dina S. El-Agamy

**Affiliations:** Department of Pharmacology and Toxicology, Faculty of Pharmacy, Mansoura University, Mansoura 35516, Egypt; xr.2@hotmail.com (S.H.A.); manargamal@mans.edu.eg (M.G.H.)

**Keywords:** ivabradine, TAA, PI3K/AKT/mTOR, Bax/Bcl-2/caspase-3, TGF-β

## Abstract

**Background**: Hepatic fibrosis and its progressive form, liver cirrhosis, are dangerously recognized complications of liver injury with limited treatment options. This study evaluated the hepatoprotective effects of ivabradine on thioacetamide (TAA)-induced hepatic fibrosis in rats. **Methods**: Rats were divided into five groups with 10 rats/group and treated as follows: normal, where rats received 0.5% CMC-Na solution orally; ivabradine control, where rats received only ivabradine (20 mg/kg, once daily, orally) for 6 weeks; TAA, where rats received an intraperitoneal (i.p.) injection of TAA (200 mg/kg) thrice weekly for 6 weeks and daily oral 0.5% CMC-Na solution, and two ivabradine + TAA groups, where two doses of ivabradine were tested. Low (10 mg/kg) and high (20 mg/kg) doses of ivabradine were orally given once daily to each group for 6 weeks concurrently with TAA injection. **Results**: TAA caused marked elevations in liver enzymes, increased MDA, depletion of antioxidant defenses, activation of NF-κB p65 and pro-inflammatory cytokines, dysregulation of apoptotic markers, and upregulation of the PI3K/AKT/mTOR and TGF-β pathways, accompanied by extensive collagen deposition. Ivabradine produced dose-dependent improvements in biochemical markers of liver function, restored oxidant/antioxidant balance, suppressed NF-κB p65/TNF-α, normalized Bax/Bcl-2/caspase-3 expression, and inhibited PI3K/AKT/mTOR as well as TGF-β signaling, leading to significant attenuation of fibrosis. **Conclusions**: The current findings indicate that ivabradine exerts potent antioxidant, anti-inflammatory, anti-apoptotic, and antifibrotic actions against TAA-induced hepatic fibrosis. Future clinical studies are recommended to determine whether these protective effects translate to patients with chronic liver disease.

## 1. Introduction

Liver fibrosis is a multifaceted inflammatory condition that can advance to cirrhosis and hepatocellular carcinoma. It represents a wound-healing response to hepatic injury, marked by excessive accumulation of extracellular matrix (ECM) components around the sinusoidal cell layer within the space of Disse. An imbalance between ECM production and its breakdown results in the progressive buildup of fibrotic tissue [1].

Thioacetamide (TAA) is a sulfur-containing compound that is known for its hepatic fibrotic and cirrhotic detrimental outcomes, which has encouraged researchers to use it in toxicological animal studies [2]. TAA is activated through the hepatic CYP450 2E1 enzyme to TAA-S-oxide and TAA-S-dioxide. The latter induces oxidative stress and lipid peroxidation of the hepatocellular membrane, leading to activation of hepatic stellate cells (HSCs) to obtain a myofibroblast-like phenotype, fibrosis, and cirrhosis [3].

In addition to oxidative stress, the PI3K/AKT/mTOR signaling axis plays a pivotal role in hepatic fibrosis development. It has been well established using a substantial amount of research that this pathway is closely linked to the activation and proliferation of HSCs and an over-accumulation of ECM [4]. Furthermore, there is increasing evidence that pharmacological inhibition as well as genetic silencing of PI3K signaling can significantly suppress hepatic stellate cell activation and restrain the deposition of extracellular matrix constituents. These interventions suppress hepatic fibrogenesis by modulating this signaling pathway, and targeting PI3K represents a promising therapeutic strategy for the management of liver fibrosis [5].

Consequently, the inhibition and suppression of the PI3K/AKT/mTOR pathway have become one of the most promising drug interventions in the prevention of hepatic fibrosis. Since there is a regulatory role that is played by PI3K/AKT/mTOR axis in cell survival, proliferation, and metabolic reprogramming, it has become an important therapeutic node, and its modulation is an attractive target towards antifibrotic drug development.

Ivabradine is a selective hyperpolarization-activated cyclic nucleotide-gated (HCN) channel blocker used to reduce heart rate and decrease hospitalization for exacerbated cases of heart failure in adult patients in addition to treating stable symptomatic heart failure caused by dilated cardiomyopathy in pediatric patients [6]. Previous studies have demonstrated that ivabradine attenuates cyclophosphamide-induced hepatotoxicity by improving liver enzymes, restoring redox balance, suppressing inflammation via the p38MAPK/NF-κB p65 and JAK1/STAT3 pathways, and modulating PI3K/AKT signaling and apoptosis [7].

Accordingly, the current investigation was therefore conducted to explore whether ivabradine has a possible protective antifibrotic effect against TAA-induced hepatic fibrosis in a rat model. To do this, a detailed assessment was conducted based on the analysis of critical biochemical markers, molecular signaling pathways, and detailed histopathological changes. The multi-level design aimed to better understand how ivabradine alleviates fibrogenesis and preserves liver structure and function.

## 2. Results

### 2.1. Impact of Ivabradine on Liver Function Biomarkers

As demonstrated in Table 1, rats administered TAA exhibited a marked rise in liver injury biomarkers. Serum levels of AST, ALT, and ALP increased by approximately 3.2-, 2.9-, and 5.7-fold, respectively, when compared with the normal control group, confirming the successful induction of hepatic damage. The low-dose ivabradine regimen significantly attenuated TAA-induced alterations, resulting in reductions of 32.8% in AST, 38.3% in ALT, and 36.7% in ALP relative to TAA-intoxicated rats. Moreover, rats receiving the high dose of ivabradine showed even greater protection, with AST, ALT, and ALP levels decreasing by 47%, 51.6%, and 55.2%, respectively, compared to the TAA-only group.

### 2.2. Impact of Ivabradine on Hepatic Histopathological Changes

**Figure 1:**
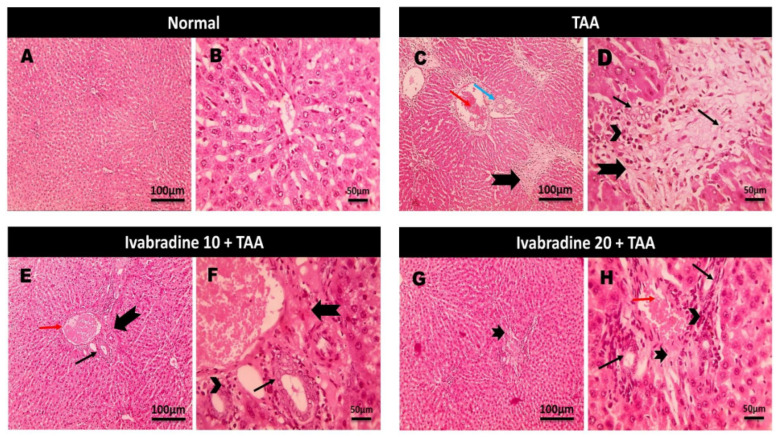
Impact of ivabradine on hepatic H&E changes. Microscopic pictures of H&E-stained liver sections. (**A**,**B**) Normal group showing normal arrangement of hepatocytes, normal central veins, portal areas, and sinusoids. (**C**,**D**) TAA group showing severe portal lesions, including fibrosis (thick black arrow), congestion (red arrow), edema (blue arrow), mononuclear cell infiltration (arrowhead), and bile ductule hyperplasia (thin black arrow). Daily administration of low (**E**,**F**) and high doses of ivabradine (**G**,**H**) induced a significant amelioration of TAA-induced histopathological changes in the liver in a dose-dependent manner.

Microscopic pictures of H&E-stained liver sections are shown in Figure 1. The normal group showed a normal arrangement of hepatocytes, normal central veins, portal areas, and sinusoids. The TAA group exhibited severe portal lesions, including fibrosis, congestion, edema, mononuclear cell infiltration, and bile ductule hyperplasia. Daily treatment with both low and high doses of ivabradine markedly improved TAA-induced histopathological alterations in the liver. The protective effect was clearly dose-dependent.

**Figure 2:**
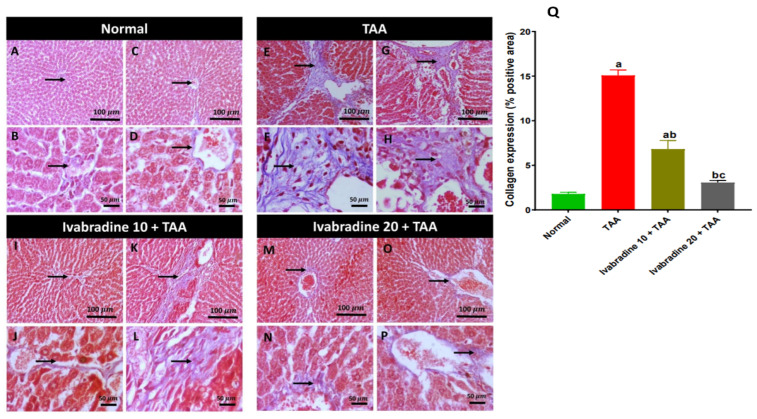
Impact of ivabradine on hepatic collagen deposition evaluated by Masson’s trichrome staining. Hepatic specimen stained with Masson’s trichrome stain for the evaluation of liver fibrosis. (**A**–**D**) Normal group showing normal deposition of collagen around blood vessels (portal or central vein). (**E**–**H**) TAA group showing dense bluish portal collagen deposition extending and separating the hepatic parenchyma. Administration of low dose of ivabradine (**I**–**L**) showed either fine bridging collagen fibrils or mild periportal collagen deposition focally extending and replacing the surrounding hepatocytes, while administering high dose of ivabradine (**M**–**P**) revealed low perivascular collagen deposition. Arrows = positive collagen deposition. Image magnification: 100× = bar 100 μm and 400× = bar 50 μm. (**Q**) effects of ivabradine on collagen deposition in the liver. Data are presented as mean ± SEM (n = 10). ^a^, ^b^, and ^c^ *p* < 0.05 significantly different compared to normal, TAA, and ivabradine 10 + TAA, respectively, using one-way ANOVA followed by the Tukey–Kramer multiple comparisons post hoc test.

Hepatic collagen deposition evaluated by Masson’s trichrome staining of different treatment groups is represented in Figure 2. The normal group showed a normal deposition of collagen around blood vessels (portal or central vein). The TAA group showed dense bluish portal collagen deposition extending and separating the hepatic parenchyma. Administration of a low dose of ivabradine showed either fine bridging collagen fibrils or mild periportal collagen deposition focally extending and replacing the surrounding hepatocytes, while administering a high dose of ivabradine revealed low perivascular collagen deposition. Rats intoxicated with TAA exhibited a pronounced increase in hepatic collagen deposition, showing approximately an 8.4-fold elevation compared to normal controls. Administration of ivabradine significantly attenuated this fibrotic response in a dose-dependent manner. Treatment with the low dose reduced collagen accumulation by approximately 55%, while the high dose produced a more substantial reduction of around 80% relative to the TAA group, which reveals a dose-dependent effect (Figure 2Q).

### 2.3. Impact of Ivabradine on Hepatic TGF-β Expression

**Figure 3:**
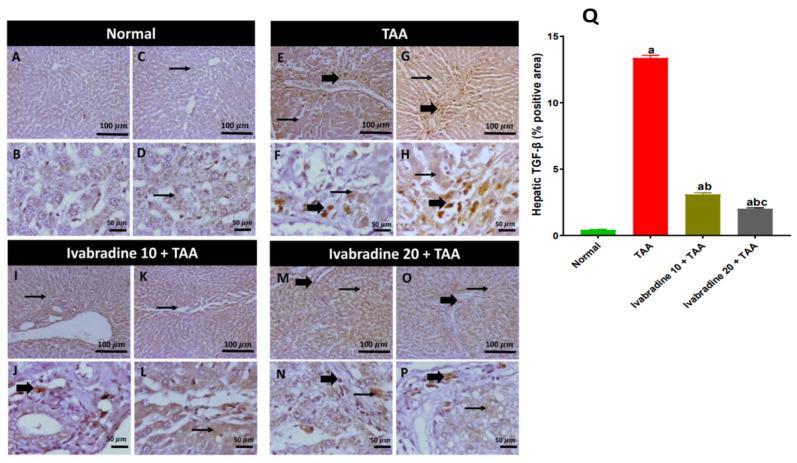
Impact of ivabradine on hepatic TGF-β expression. Representative TGF-β expression and localization in hepatic sections of different treatment groups. (**A**–**D**) Control group (low- and high-power view) showing negative-to-low cytoplasmic expression in hepatocytes. (**E**–**H**) TAA group (low- and high-power view) showing diffuse moderate-to-high cytoplasmic expression in hepatocytes and invading inflammatory cells. (**I**–**L**) Low ivabradine (low- and high-power view) showing low-to-mild faint cytoplasmic expression in hepatocytes and invading inflammatory cells. (**M**–**P**) High ivabradine (low- and high-power view) showing mild faint cytoplasmic expression in hepatocytes with scattered positivity in invading inflammatory cells. Thin arrows = positive hepatocytes, thick arrows = positive inflammatory cells. Image magnification: 100× = bar 100 μm, 400× = bar 50 μm; (**Q**) TGF-β percentage expression in the hepatic sections of different groups. Data are presented as mean ± SEM (n = 10). ^a^, ^b^, and ^c^ *p* < 0.05 significantly different compared to normal, TAA, and ivabradine 10 + TAA, respectively, using one-way ANOVA followed by the Tukey–Kramer multiple comparisons post hoc test.

Hepatic expression of TGF-β was profoundly elevated in the TAA control group, exhibiting an approximate 31-fold increase relative to normal controls. Oral administration of ivabradine at 10 mg/kg and 20 mg/kg markedly attenuated this increase in a dose-dependent manner, resulting in reductions of 70.6% and 84.9%, respectively, compared to the TAA group (Figure 3).

### 2.4. Impact of Ivabradine on Hepatic α-SMA Expression

**Figure 4:**
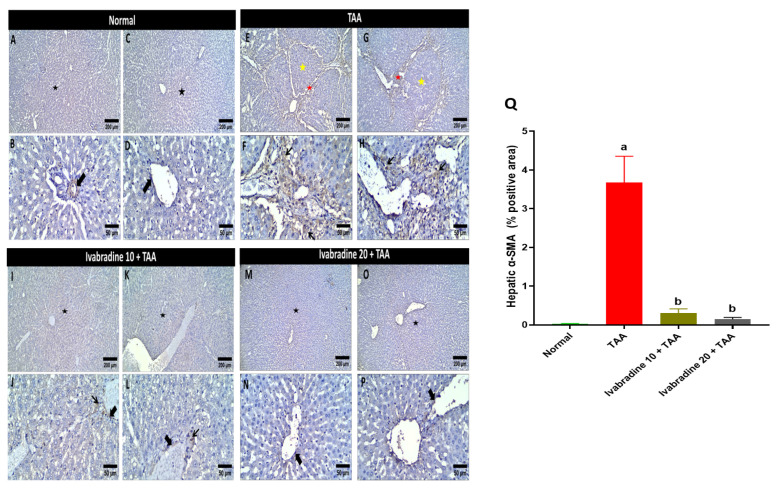
Impact of ivabradine on hepatic α-SMA expression. Representative α-SMA expression and localization in hepatic sections of different treatment groups. (**A**–**D**) Control group (low- and high-power view) showing preserved hepatic architecture without nodule formation (black star). Negative cytoplasmic expression of α-SMA is observed in hepatocytes, with positive brownish immunostaining restricted to the blood vessel walls (thick arrows). (**E**–**H**) TAA group (low- and high-power view) showing significant loss of hepatic architecture and extensive nodule formation (yellow star). Marked bridging fibrosis with intense brownish staining is evident (red star), along with diffuse cytoplasmic expression of α-SMA in activated stellate cells (thin arrows). (**I**–**L**) Low ivabradine (low- and high-power view) showing improvement and protective antifibrotic effects; the hepatic architecture is restored, showing only low scattered cytoplasmic expression in the pericentral area while maintaining normal staining of blood vessel walls. (**M**–**P**) High ivabradine (low- and high-power view) showing near-normal restoration of hepatic architecture with a significant reduction in α-SMA expression, which is primarily limited to the blood vessel walls, similar to the control group. Image magnification: 100× = bar 200 μm, 400× = bar 50 μm; (**Q**) α-SMA percentage expression in the hepatic sections of different groups. Data are presented as mean ± SEM (n = 10). ^a^ and ^b^ *p* < 0.05 significantly different compared to normal and TAA, respectively, using one-way ANOVA followed by the Tukey–Kramer multiple comparisons post hoc test.

The percentage of hepatic α-SMA-positive cells in TAA-injected rats was significantly greater than that of the normal rats by 146.8-fold. Oral administration of ivabradine at 10 mg/kg and 20 mg/kg substantially reduced the hepatic expression of α-SMA in TAA-injected rats by 91.7% and 96.1%, respectively (Figure 4).

### 2.5. Impact of Ivabradine on Hepatic COL1A1 Expression

**Figure 5:**
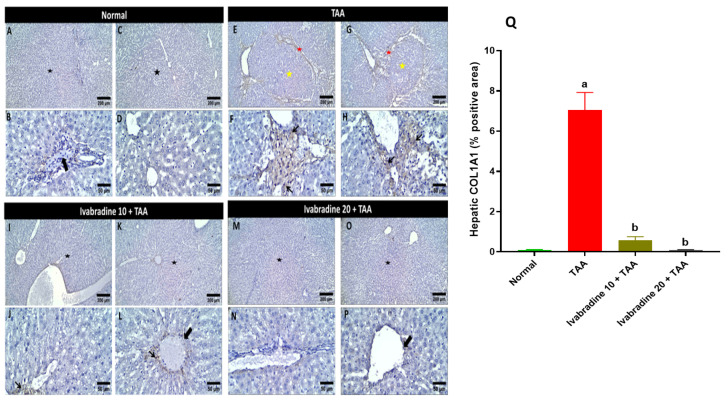
Impact of ivabradine on hepatic α-SMA expression. Representative COL1A1 expression and localization in hepatic sections of different treatment groups. (**A**–**D**) Control group (low- and high-power view) showing preserved hepatic architecture without nodule formation (black star). Negative cytoplasmic expression of COL1A1 is observed in hepatocytes, with positive brownish immunostaining restricted to the blood vessel walls (thick arrows). (**E**–**H**) TAA group (low- and high-power view) showing significant loss of hepatic architecture and extensive nodule formation (yellow star). Marked bridging fibrosis with intense brownish staining is evident (red star), along with diffuse cytoplasmic expression of COL1A1 in activated stellate cells (thin arrows). (**I**–**L**) Low ivabradine (low- and high-power view) showing improvement and protective antifibrotic effects; the hepatic architecture is restored, showing only low scattered cytoplasmic expression in the pericentral area while maintaining normal staining of blood vessel walls. (**M**–**P**) High ivabradine (low- and high-power view) showing near-normal restoration of hepatic architecture with a significant reduction in COL1A1 expression, which is primarily limited to the blood vessel walls, similar to the control group. Image magnification: 100× = bar 200 μm, 400× = bar 50 μm; (**Q**) COL1A1 percentage expression in the hepatic sections of different groups. Data are presented as mean ± SEM (n = 10). ^a^ and ^b^ *p* < 0.05 significantly different compared to normal and TAA, respectively, using one-way ANOVA followed by the Tukey–Kramer multiple comparisons post hoc test.

Injection of TAA promptly elevated the hepatic expression of COL1A1 by 70-fold compared to normal rats. Administration of both doses of ivabradine (10 and 20 mg/kg) significantly diminished the hepatic expression of COL1A1 by 92% and 98.5%, respectively, compared to TAA-injected rats (Figure 5).

### 2.6. Impact of Ivabradine on Hepatic Oxidative/Antioxidant Capacity

**Figure 6:**
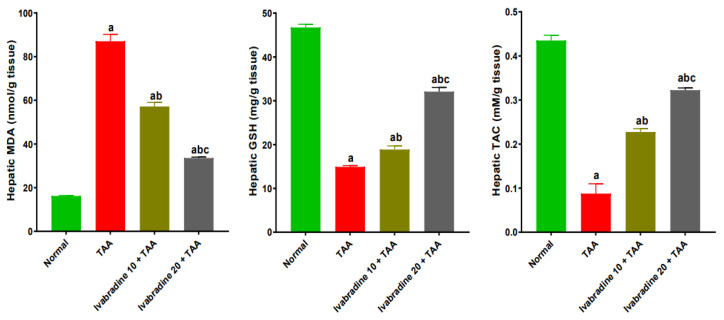
Impact of ivabradine on hepatic oxidative/antioxidant capacity. Data are presented as mean ± SEM (n = 10). ^a^, ^b^, and ^c^ *p* < 0.05 significantly different compared to normal, TAA, and ivabradine 10 + TAA, respectively, using one-way ANOVA followed by the Tukey–Kramer multiple comparisons post hoc test.

Rats exposed to TAA exhibited a pronounced increase in hepatic MDA levels, approximately 5.3-fold higher than normal controls. Treatment with ivabradine significantly attenuated this effect in a dose-dependent manner, reducing MDA content by roughly 34.3% at the low dose and 61.4% at the high dose relative to the TAA group. Conversely, TAA administration caused a marked depletion of hepatic GSH, with levels decreasing by approximately 68.1% compared to normal controls. Oral administration of ivabradine restored GSH levels significantly, increasing them by approximately 1.3-fold and 2.2-fold for the low and high doses, respectively, relative to TAA-injected rats. Similarly, TAC was markedly suppressed in TAA-injected rats, showing an 81.4% reduction compared with normal controls. Ivabradine treatment effectively enhanced hepatic TAC in a dose-dependent fashion, resulting in increases of approximately 2.6-fold and 3.7-fold at low and high doses, respectively, compared to the TAA group (Figure 6).

### 2.7. Impact of Ivabradine on Hepatic NF-κB p65/TNF-α Expressions

**Figure 7:**
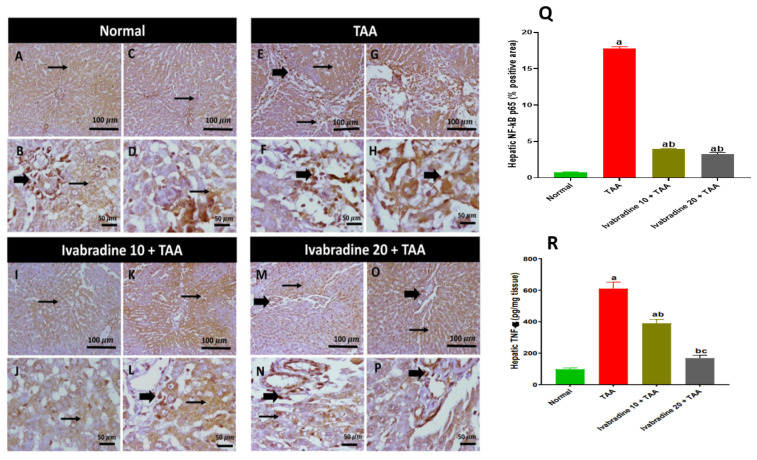
Impact of ivabradine on the hepatic expressions of NF-κB p65/TNF-α. Representative NF-κB p65 expression and localization in hepatic sections of different treatment groups. (**A**–**D**) Normal group (low- and high-power view) showing faint cytoplasmic and nuclear expression in hepatocytes. (**E**–**H**) TAA group (low- and high-power view) showing diffuse high cytoplasmic expression in hepatocytes with nuclear expression in surrounding periportal invading inflammatory cells. (**I**–**L**) Low ivabradine (low- and high-power view) showing moderate faint-to-strong cytoplasmic expression with scattered nuclear positivity in invading inflammatory cells. (**M**–**P**) High ivabradine (low- and high-power view) showing mild-to-moderate cytoplasmic expression in hepatocytes. Thin arrows = positive hepatocytes, thick arrows = positive inflammatory cells. Image magnification: 100× = bar 100 μm, 400× = bar 50 μm; (**Q**) NF-κB p65 percentage expression in the hepatic sections of different treatment groups; (**R**) level of TNF-α in hepatic tissue. Data are presented as mean ± SEM (n = 10). ^a^, ^b^, and ^c^ *p* < 0.05 significantly different compared to normal, TAA, and ivabradine 10 + TAA, respectively, using one-way ANOVA followed by the Tukey–Kramer multiple comparisons post hoc test.

The percentage of hepatic NF-κB p65-positive cells in the TAA-injected group was significantly greater than that of the normal control by 23.6-fold. Oral treatment with ivabradine at 10 mg/kg and 20 mg/kg substantially suppressed hepatic NF-κB p65 expression in TAA-injected rats. The reductions were approximately 79.2% and 81.6%, respectively, compared with the TAA group (Figure 5). TAA significantly increased the hepatic expression of TNF-α by 6.2-fold. Low- and high-dose ivabradine significantly reduced hepatic TNF-α expression by 36% and 72.3%, respectively (Figure 7).

### 2.8. Impact of Ivabradine on Hepatic PI3K, pPI3K, AKT, pAKT, and mTOR Expressions

**Figure 8:**
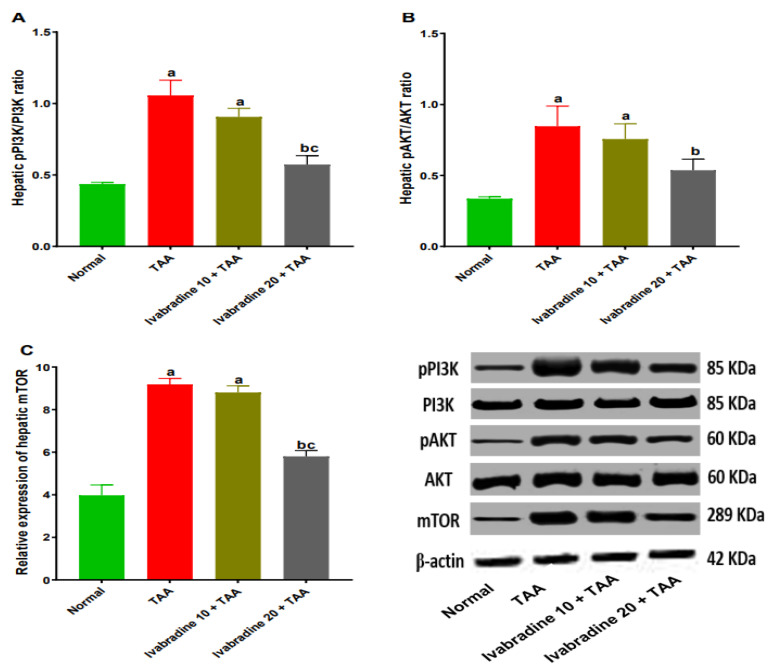
(**A**–**C**) Impact of ivabradine on hepatic PI3K, pPI3K, AKT, pAKT, and mTOR expressions. Data are presented as mean ± SEM (n = 3). ^a^, ^b^, and ^c^ *p* < 0.05 significantly different compared to normal, TAA, and ivabradine 10 + TAA, respectively, using one-way ANOVA followed by the Tukey–Kramer multiple comparisons post hoc test.

Intoxication with TAA elicited a marked elevation in hepatic pPI3K/PI3K and pAKT/AKT ratios by 2.4- and 2.5-fold, respectively, compared to normal rats. Additionally, TAA-injected rats revealed a significant increase in hepatic expression of mTOR by 2.3-fold compared to normal rats. Concurrent administration of low-dose ivabradine non-significantly reduced hepatic pPI3K/PI3K and pAKT/AKT ratios and mTOR expression by 14.2%, 10.7%, and 3.3%, respectively, compared to TAA-intoxicated rats. However, administration of a higher dose of ivabradine significantly reduced hepatic pPI3K/PI3K and pAKT/AKT ratios and mTOR expression by 46%, 37%, and 37%, respectively, compared to TAA-intoxicated rats (Figure 8).

### 2.9. Impact of Ivabradine on the Hepatic Levels of Bax and Caspase-3 Expressions

**Figure 9:**
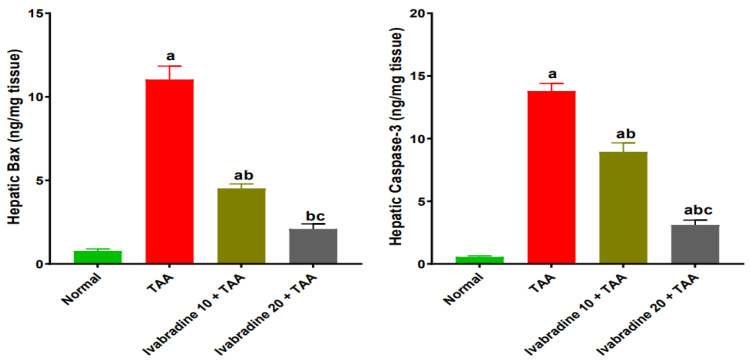
Impact of ivabradine on hepatic levels of Bax and caspase-3. Data are presented as mean ± SEM (n = 10). ^a^, ^b^, and ^c^ *p* < 0.05 significantly different compared to normal, TAA, and ivabradine 10 + TAA, respectively, using one-way ANOVA followed by the Tukey–Kramer multiple comparisons post hoc test.

Injection of TAA demonstrated a prompt increase in hepatic expressions of Bax and caspase-3 by 14.1, and 24.6-fold, respectively, compared to normal rats. A low dose of ivabradine showed a significant reduction in hepatic expressions of Bax and caspase-3 by 59%, and 35.5%, respectively, compared to TAA-injected rats. Moreover, administration of ivabradine at a higher dose significantly diminished the hepatic expressions of Bax and caspase-3 by 81%, and 77.4%, respectively, compared to TAA-injected rats (Figure 9).

### 2.10. Impact of Ivabradine on Hepatic Bcl-2 Expression

**Figure 10:**
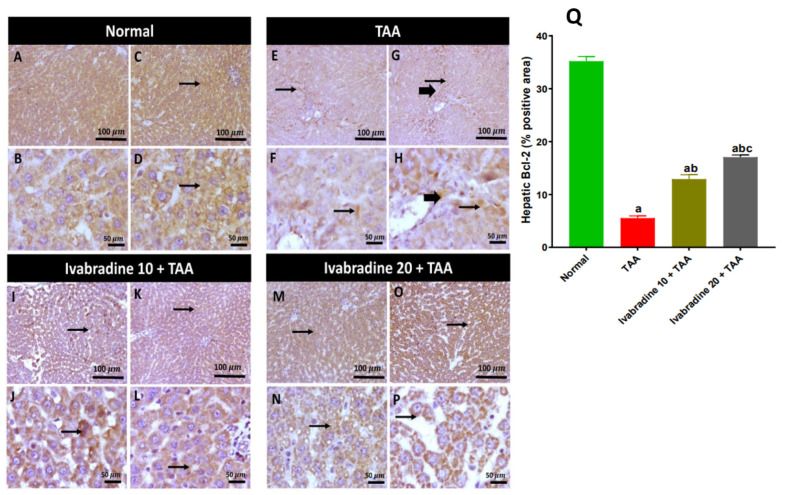
Impact of ivabradine on hepatic Bcl-2 expression. Representative Bcl-2 expression and localization in hepatic sections of different treatment groups. (**A**–**D**) Normal group (low- and high-power view) showing extensive cytoplasmic expression in hepatocytes. (**E**–**H**) TAA group (low- and high-power view) showing low-to-mild cytoplasmic expression in hepatocytes with low positivity in invading inflammatory cells. (**I**–**L**) Low ivabradine (low- and high-power view) showing moderate immunopositive cytoplasmic stained hepatocytes. (**M**–**P**) High ivabradine (low- and high-power view) showing moderate-to-high cytoplasmic expression in hepatocytes. Thin arrows = positive hepatocytes, thick arrow = positive inflammatory cells. Image magnification: 100× = bar 100μm, 400× = bar 50 μm; (**Q**) Bcl-2 percentage expression in the hepatic sections of different treatment groups; data are presented as mean ± SEM (n = 10). ^a^, ^b^, and ^c^ *p* < 0.05 significantly different compared to normal, TAA, and ivabradine 10 + TAA, respectively, using one-way ANOVA followed by the Tukey–Kramer multiple comparisons post hoc test.

TAA administration caused a significant suppression of hepatic Bcl-2 expression, with levels decreasing by approximately 84.3% compared to normal controls. Oral treatment with ivabradine at 10 mg/kg and 20 mg/kg markedly restored Bcl-2 expression in a dose-dependent manner, resulting in increases of approximately 2.3-fold and 3.1-fold, respectively, relative to the TAA group (Figure 10).

## 3. Discussion

Liver fibrosis is a major public health issue that can lead to liver cancer, cirrhosis, and even death. The hepatotoxin TAA is known to cause nearly irreversible fibrosis in rodents that is comparable to that in humans. TAA-induced liver fibrosis is more effective than other experimental models for testing possible antifibrotic medications [8].

Cytosolic enzymes, AST, ALT, and ALP, are well-recognized diagnostic markers of hepatic cellular integrity. Our results revealed significant high levels of these markers in the TAA group, indicating hepatic injury and deterioration as well as suppression of the liver’s synthesizing and detoxifying processes. The histopathology of the liver specimens confirmed the necrotic, inflammatory and fibrotic changes in the hepatic tissue in the case of TAA administration. These results were consistent with earlier research [9,10,11,12]. Notably, ivabradine prevented TAA’s deleterious effects on the hepatocytes, as there was a significant amelioration of the liver function biomarkers as well as a noticeable improvement in TAA-induced histopathological lesions. It is worth noting that ivabradine has shown hepatoprotective activity against acute cyclophosphamide-induced hepatotoxicity [7].

The reactive metabolites of TAA are strong promoters of the generation of reactive oxygen species (ROS) that eventually result in exhaustion of the antioxidant defense system of the hepatic tissue and damaged GSH homeostasis. Our data confirmed the impaired oxidant/antioxidant balance after TAA exposure, as there was increased hepatic MDA, a principal end-product of lipid peroxidation, and reduced endogenous antioxidants, including GSH and TAC, which is consistent with previous studies [13,14,15]. Ivabradine significantly reversed these alterations in a dose-dependent manner, reducing MDA and restoring GSH and TAC levels, indicating its ability to counteract oxidative injury, a finding that is in harmony with earlier research that showed a potential antioxidant effect of ivabradine in the case of convulsion and dementia [16,17]. Although traditionally considered a selective HCN channel blocker for heart rate reduction, accumulating evidence shows that ivabradine can exert direct antioxidant effects. In vascular and cardiac tissues, ivabradine reduces ROS formation and enhances endogenous antioxidant defenses by suppressing NOX2 activity and improving mitochondrial efficiency [18]. By attenuating oxidative stress, ivabradine may interrupt the vicious cycle between ROS generation, NF-κB p65 activation, and HSC stimulation, thereby limiting fibrogenesis progression.

The interplay between ROS and inflammation has been increasingly recognized as a central mechanism in the pathogenesis of numerous diseases, including cancer, neurodegenerative disorders, cardiovascular diseases, liver diseases, autoimmune conditions, and several other pathological states [19]. ROS promote the activation of key signaling mediators, including the transcription factor NF-κB p65, which subsequently drives the production of pro-inflammatory cytokines, such as IL-1β, IL-6, and TNF-α, thereby amplifying inflammatory responses and contributing to tissue damage [20]. Hereby, our data revealed that TAA administration significantly increased hepatic NF-κB p65 expression and TNF-α levels, consistent with prior reports demonstrating NF-κB p65 activation as a hallmark of TAA-induced liver damage [21,22,23]. Ivabradine markedly suppressed NF-κB p65 and TNF-α expression. These findings align with earlier studies showing the ability of ivabradine to reduce inflammatory signaling by inhibiting NF-κB p65 nuclear translocation in cardiovascular models [24,25].

The PI3K/AKT/mTOR signaling pathway plays a central role in hepatocyte survival, HSC activation, and ECM deposition [26]. Numerous investigations have revealed the pivotal role of this pathway in the progression of hepatic fibrosis. The close relation between PI3K/AKT/mTOR and the activation and proliferation of HSCs and subsequent ECM deposition have been well documented [13]. Additionally, former reports showed that inhibition of PI3K signaling results in attenuation of hepatic fibrosis [5]. In the current study, TAA markedly increased pPI3K/PI3K and pAKT/AKT ratios and upregulated mTOR expression, confirming pathway activation. These results are in line with the results of earlier investigations [13,27,28]. Administration of ivabradine significantly suppressed activation of this pathway in a dose-dependent manner. Previous studies have reported that ivabradine protected rats against myocardial infarction through reinforcing autophagy via inhibiting the PI3K/AKT/mTOR pathway [29]. By downregulating this survival and growth-promoting cascade, ivabradine likely limits both hepatocyte stress responses and HSC proliferation.

Additionally, PI3K/AKT activation enhances NF-κB p65 activity and increases TGF-β production, forming a signaling axis that drives fibrosis [27,30]. Therefore, ivabradine’s suppression of this pathway may explain concurrent reductions in NF-κB p65, TGF-β, and collagen deposition. TGF-β is the master profibrotic cytokine responsible for HSC activation and excessive ECM deposition in chronic liver injury [31]. Expectedly, the TAA group showed marked increases in TGF-β expression and Masson’s-trichrome-positive collagen fibers, which is consistent with the results of former studies [11,28,32]. Interestingly, ivabradine reduced TGF-β expression and markedly attenuated collagen deposition in a dose-dependent manner in the case of cardiac dysfunction [33,34,35]. The improvement in histological fibrosis corresponds directly to molecular findings, suggesting that ivabradine interferes with several upstream drivers of TGF-β, including oxidative stress, NF-κB p65 activation, and PI3K/AKT/mTOR signaling.

The enhanced oxidative and inflammatory responses in the liver following TAA exposure stimulate apoptotic cell death due to the disruption between anti-apoptotic and pro-apoptotic mediators [11]. Our results showed that TAA significantly increased the pro-apoptotic markers Bax and caspase-3 while reducing the anti-apoptotic protein Bcl-2, consistent with mitochondrial-dependent apoptosis documented in TAA models [12,13]. Excessive apoptosis contributes not only to hepatocyte loss but also to fibrosis, as apoptotic bodies stimulate HSC activation [36,37]. Ivabradine shifted the apoptotic balance toward cell survival by reducing Bax and caspase-3 and enhancing Bcl-2 expression. Similar anti-apoptotic effects of ivabradine have been described in chronic viral myocarditis and isoprenaline-induced heart failure models, where the drug reduced mitochondrial membrane depolarization and inhibited caspase activation [38,39]. This protective effect may be partially mediated through suppression of oxidative stress and inflammatory mediators, both of which trigger hepatocyte apoptosis. By preserving hepatocyte integrity, ivabradine likely reduces the apoptotic stimuli that propagate fibrogenesis. Collectively, the suppression of PI3K/AKT/mTOR signaling appears to represent a central upstream event through which ivabradine orchestrates its antioxidant, anti-inflammatory, anti-apoptotic, and antifibrotic effects.

## 4. Materials and Methods

### 4.1. Animals

The Urology and Nephrology Centre, Mansoura University, supplied 50 male Sprague–Dawley rats (weight 200 ± 20 g). Before being used, the animals were kept under normal environmental conditions and allowed a two-week period to acclimatize in a normal laboratory environment. All animal experiments complied with the institutional guidelines of the Mansoura University Animal Care and Use Committee, and the Faculty of Pharmacy Research Ethics Committee gave its ethical approval (approval no.: MU-ACUC (PHARM.PhD.23.01.14)). The experiment was done according to the National Institute of Health (NIH) guidelines Care and Use of Laboratory Animals (publication no. 85-23, revised 1985). The authors complied with the ARRIVE 2.0 guidelines.

### 4.2. Drugs and Chemicals

Ivabradine tablets (Procoralan^®^) were utilized (Servier Egypt Industries Limited, Cairo, Egypt) and suspended in sodium carboxymethyl cellulose (CMC-Na) to be taken orally. TAA was purchased from Alfa Aesar (Ward Hill, MA, USA). All the other chemicals used in the study were of high grade and analytical purity.

### 4.3. Experimental Protocol

Rats were divided into 5 groups with 10 rats/group and treated as follows: normal, where rats received 0.5% CMC-Na solution, orally; ivabradine control, where rats received only ivabradine (20 mg/kg, once daily, orally) for 6 weeks; TAA, where rats received intraperitoneal (i.p.) injection of TAA (200 mg/kg) thrice weekly for 6 weeks [8] and daily oral 0.5% CMC-Na solution and two ivabradine + TAA groups, where two doses of ivabradine were tested. Low (10 mg/kg) and high (20 mg/kg) doses of ivabradine were orally given once daily to each group for 6 weeks concurrently with TAA injection. The doses of ivabradine were selected based on previous studies [16,18].

The animals were anesthetized by the use of thiopental sodium (40 mg/kg, i.p.) 24 h following the last administration of TAA. Capillary tubes were used to collect blood samples by means of the retro-orbital venous plexus. Blood obtained was left to clot at room temperature and then centrifuged with 4000 rpm at 15 min to get a clear serum. Separated serum was stored by being aliquoted and maintained at −80 °C until required to conduct the consequent biochemical assessments.

After liver excision, the liver was cut into separate lobes. The left hepatic lobe was immediately fixed in neutral-buffered formalin, 10% (*v*/*v*), followed by processing of the liver to undergo histopathological and immunohistochemical analyses. A 10% (*w*/*v*) tissue homogenate was made by weighing the right lobe and subsequently homogenizing it in ice-cold phosphate-buffered saline (PBS). The homogenate was centrifuged at 4000 rpm for 15 min at 4 °C. The supernatant was collected, aliquoted, and stored at −80 °C until further biochemical and ELISA analysis. The remaining part of the liver was quickly snap-frozen and put away to be used in Western blotting.

### 4.4. Biochemical Evaluation of Biomarkers of Liver Function

Alanine aminotransferase (ALT), aspartate aminotransferase (AST) and alkaline phosphatase (ALP) serum levels were measured using commercially available diagnostic kits (Bio-Diagnostic, Giza, Egypt), in accordance with the guidelines provided by the manufacturer.

### 4.5. Biochemical Evaluation of Hepatic Oxidative/Antioxidant Capacity

Malondialdehyde (MDA), reduced glutathione (GSH), and total antioxidant capacity (TAC) concentrations in liver homogenates were measured using Bio-Diagnostic assay kits, which are commercially available (Giza, Egypt).

### 4.6. Western Blot Analysis of Hepatic Phosphoinositide 3-Kinase (PI3K), Phosphorylated Phosphoinositide 3-Kinase (pPI3K), Protein Kinase B (AKT), Phosphorylated Protein Kinase B (pAKT), and Mammalian Target of Rapamycin (mTOR) Expression

Approximately 100 mg of liver tissue was homogenized in 1 mL of TriFast reagent (Peqlab, VWR, Soborg, Denmark) to extract total protein. Protein concentration was then determined using the Bradford assay method. Equal quantities of protein (30 µg per lane) were separated on SDS-PAGE, with electrophoresis performed initially at 75 V and then increased to 125 V for a total running period of about 2 h. Following separation, proteins were transferred onto a Hybond™ nylon membrane (GE Healthcare, Chicago, IL, USA) using a TE62 Standard Transfer Tank equipped with a cooling chamber (Hoefer Inc., Bridgewater, MA, USA) and blotting buffer containing 25 mM Tris, 0.15 M NaCl, and 0.1% Tween-20 (pH 7.4) [40]. To prevent nonspecific antibody attachment, the membrane was blocked with 5% skim milk prepared in TBST buffer (4 mM Tris base, 100 mM NaCl, 0.05% Tween-20, pH 7.5) for 1 h at room temperature. The blots were then incubated overnight at 4 °C with gentle agitation using the primary antibodies PI3K p85 (Cell Signaling, Danvers, MA, USA, #4292, 85 kDa, 1:1000), phosphorylated PI3K p85/p55 (Tyr467/Tyr199/Tyr464) (Invitrogen, Carlsbad, CA, USA, #PIPA5121306, 85 kDa, 1:1000), AKT (Cell Signaling, #9272, 60 kDa, 1:1000), phosphorylated AKT (Ser473) (Cell Signaling, #9271, 60 kDa, 1:1000), and mTOR (Cell Signaling, #2972, 289 kDa, 1:1000). After primary antibody incubation, the membranes were washed repeatedly at room temperature for approximately 60 min using five fresh changes of wash buffer. The blots were then exposed to an HRP-conjugated anti-rabbit IgG secondary antibody (R&D Systems, Minneapolis, MN, USA, #HAF008, 1:1000) for 1 h at room temperature. Protein bands were visualized using WesternBright™ ECL detection reagent (Advansta, San Jose, CA, USA, #K-12045) and captured with an Azure 600 Imaging System (Azure Biosystems, Dublin, CA, USA). Densitometric analysis of band intensity was performed using a gel documentation unit (Geldoc-it, UVP, London, UK) with the Totallab software (version 1.0.1). Band intensities were normalized to β-actin (Abcam, Cambridge, UK, #ab8227, 42 kDa, 1:1000) to control for protein loading and transfer efficiency. All Western blot analyses were conducted on three independent biological replicates per experimental group to ensure reproducibility.

### 4.7. Biochemical Evaluation of Hepatic Tumor Necrosis Factor-Alpha (TNF-α), Bcl-2-Associated X Protein (Bax), and Caspase-3 Expression

Hepatic contents of TNF-α, Bax, and caspase-3 were measured using ELISA kits obtained from Cusabio (Houston, TX, USA, #CSB-E11987r; #CSB-E08857r) and Biovision (Milpitas, CA, USA, #E4513-100), in agreement with the measures defined by the producers.

### 4.8. Histopathological and Immunohistochemical Examination

Formalin-fixed liver tissues were embedded in paraffin wax, sectioned, and stained using Hematoxylin and Eosin (H&E) as well as Masson’s trichrome (MT) to assess tissue architecture and detect collagen deposition indicative of fibrosis. Histological observations were carried out using a light microscope (Leica Imaging Systems, Cambridge, UK). All slides were evaluated by an expert pathologist who was blinded to the experimental grouping to eliminate bias in interpretation.

For immunohistochemical analysis, nuclear factor kappa-B p65 (NF-κB p65), B-cell lymphoma-2 (Bcl-2), transforming growth factor-β (TGF-β), α-smooth muscle actin (α-SMA), and collagen alpha-1 (I) chain (COL1A1) expression levels were examined in liver sections. Briefly, paraffin-embedded samples were deparaffinized, rehydrated, and subjected to antigen retrieval to enhance epitope exposure. The sections were incubated overnight at 4 °C with rabbit polyclonal primary antibodies, including NF-κB p65 (Invitrogen, USA, #PA5-16545), Bcl-2 (Abcam, Waltham, MA, USA, #ab194583), TGF-β (Abcam, USA, #ab215715), α-SMA (ServiceBio, Wuhan, China, #GB111364), and COL1A1 (ServiceBio, China, #GB11022-3). After thorough washing, the tissues were incubated with the appropriate secondary antibodies (Genemed Biotechnologies, San Francisco, CA, USA) for 2 h at room temperature. Visualization was achieved using diaminobenzidine (DAB) as a chromogen, and the stained tissues were subsequently examined microscopically (Leica Imaging Systems). Negative and control staining procedures were performed in parallel to rule out background staining or nonspecific antibody binding, ensuring reliability of the immunohistochemical results [41]. The immunohistochemical staining was quantified using the ImageJ software 1.54g (NIH, Bethesda, MD, USA), where five random fields per section were captured at 400× magnification. The percentage of positive area for NF-κB p65 and TGF-β was calculated and averaged for each sample.

### 4.9. Statistical Analysis

All data are expressed as the mean ± standard error of the mean (SEM). Statistical analysis, data visualization, and ELISA standard curve fitting were conducted using the GraphPad Prism software (Version 8, San Diego, CA, USA). A probability value of *p* < 0.05 was considered statistically significant. Normality of each dataset was assessed using the Shapiro–Wilk test, and homogeneity of variance was evaluated using Levene’s test. For datasets meeting these assumptions, one-way ANOVA followed by Tukey–Kramer’s post hoc test was applied, while datasets that violated these assumptions, including semi-quantitative histopathology and immunohistochemistry scores, were analyzed using the non-parametric Kruskal–Wallis test with Dunn’s post hoc comparisons.

## 5. Conclusions

In summary, this study provides evidence that ivabradine is associated with hepatoprotective and antifibrotic effects in a rat model of TAA-induced liver fibrosis. These benefits were reflected by improvements in serum liver enzymes, attenuation of oxidative stress, and modulation of inflammatory and apoptotic pathways. Ivabradine treatment was associated with alterations in multiple signaling cascades, including NF-κB-p65/TNF-α, PI3K/AKT/mTOR, and the Bax/Bcl-2 axis, in a dose-dependent manner. Collectively, these findings suggest a multi-targeted association between ivabradine and attenuation of hepatic injury and fibrogenesis. Further clinical studies are warranted to explore whether these observations translate into therapeutic benefits for patients with chronic liver disease.

### Study Limitations

Despite the promising findings, several limitations should be acknowledged. First, the study was conducted in a single experimental model of hepatic fibrosis (TAA-induced) in rats, which may not fully replicate the complexity and heterogeneity of human chronic liver disease. Second, only two doses of ivabradine were evaluated, and pharmacokinetic parameters were not assessed, limiting conclusions regarding optimal dosing and systemic exposure. Additionally, the study duration was restricted to six weeks, which may not reflect long-term therapeutic efficacy or safety. Mechanistically, the study relied primarily on protein expression analyses to infer involvement of multiple signaling pathways without employing specific pathway inhibitors, activators, or genetic modulation to establish causal relationships. As such, the findings should be interpreted as associative rather than definitive mechanistic evidence. Finally, translation of these observations to clinical settings should be approached cautiously, as species differences, comorbidities, and inter-individual variability in humans may influence therapeutic outcomes.

## Figures and Tables

**Table 1:** Impact of ivabradine on liver function biomarkers. Data are presented as mean ± SE (n = 10). ^a^, ^b^, and ^c^ *p* < 0.05 significantly different compared to normal, TAA, and ivabradine 10 + TAA, respectively, using one-way ANOVA followed by the Tukey–Kramer multiple comparisons post hoc test.

Experimental Group	AST (IU/L)	ALT (IU/L)	ALP (IU/L)
Normal	157.5 ± 22.1	50.5 ± 1.9	135.5 ± 5.9
Ivabradine Control	201.0 ± 1.1	58.5 ± 0.6	140.5 ± 0.6
TAA	511.5 ± 32.9 ^a^	150.8 ± 15.1 ^a^	766.8 ± 21.9 ^a^
Ivabradine 10 + TAA	343.8 ± 7.3 ^ab^	93.0 ± 1.9 ^ab^	485.5 ± 7.1 ^ab^
Ivabradine 20 + TAA	271.3 ± 8.9 ^ab^	73.0 ± 1.8 ^b^	342.8 ± 14.9 ^abc^

## Data Availability

The original contributions presented in this study are included in the article. Further inquiries can be directed to the corresponding authors.

## Abbreviations

AKT	Protein kinase B
ALP	Alkaline phosphatase
ALT	Alanine aminotransferase
AST	Aspartate aminotransferase
α-SMA	α-Smooth muscle actin
Bax	Bcl-2-associated X protein
Bcl-2	B-cell lymphoma-2
CMC-Na	Sodium carboxymethyl cellulose
COL1A1	Collagen alpha-1 (I) chain
ELISA	Enzyme-linked immunosorbent assay
GSH	Reduced glutathione
H&E	Hematoxylin–eosin
HSCs	Hepatic stellate cells
i.p.	Intraperitoneal
MDA	Malondialdehyde
MT	Masson’s trichrome
mTOR	Mammalian target of rapamycin
NF-κB p65	Nuclear factor kappa-B p65
PBS	Phosphate-buffered saline
PI3K	Phosphoinositide 3-kinase
ROS	Reactive oxygen species
TAA	Thioacetamide
TAC	Total antioxidant capacity
TBST	Tris-buffered saline with Tween 20
TGF	Transforming growth factor
TNF	Tumor necrosis factor
